# Predictors of Diabetic Foot and Leg Ulcers in a Developing Country with a Rapid Increase in the Prevalence of Diabetes Mellitus

**DOI:** 10.1371/journal.pone.0080856

**Published:** 2013-11-06

**Authors:** Kumarasinghe A. Sriyani, Sudharshani Wasalathanthri, Priyadharshika Hettiarachchi, Shamini Prathapan

**Affiliations:** 1 Department of Health Sciences, the Open University of Sri Lanka, Nawala, Sri Lanka; 2 Department of Physiology, University of Colombo, Colombo, Sri Lanka; 3 Department of Physiology, University of Sri Jayewardenepura, Gangodawila, Nugegoda, Sri Lanka; 4 Department of Community Medicine, University of Sri Jayewardenepura, Gangodawila, Nugegoda, Sri Lanka; University of Michigan Medical School, United States of America

## Abstract

**Objective:**

To identify the socio demographic, life style and foot examination related predictors of diabetic foot and leg ulcers with a view to develop a screening tool appropriate for the use in an outpatient setting.

**Research design and methods:**

This cross sectional study included type 2 diabetes mellitus (DM) patients; 88 subjects with leg and foot ulcers and 80 non ulcer controls. Socio demographic data and life style factors were documented. Foot was examined for skin changes and structural abnormalities. Distal peripheral neuropathy was assessed by pressure sense, vibration sense and joint position sense. Multivariate analysis by logistic regression was used to determine the significant predictors in screening for foot ulcers.

**Results:**

Education of grade 6 and below (OR - 1.41, 95% CI; 1.03 - 4.68), low income (OR - 23.3, 95% CI; 1.5 - 34.0), impaired vibration sense (OR - 24.79, 95% CI; 9.3 - 66.2), abnormal monofilament test on first (OR - 1.69, 95% CI; 1.36 - 16.6), third (OR - 3.4, 95% CI; 1.1 - 10.6) and fifth (OR - 1.8, 95% CI; 1.61- 12.6) toes are found to be predictors of increased risk whereas incidental diagnosis of DM (OR - 0.03, 95% CI; 0.003 - 0.28), wearing covered shoes (OR - 0.003, 95% CI; 0.00 - 0.28), presence of normal skin color (OR - 0.01, 95% CI; 0.001 - 0.14) and normal monofilament test on first metatarsal head (OR - 0.10, 95% CI; 0.00 - 0.67) are protective factors for ulcers.

**Conclusions:**

Ten independent risk and protective factors identified in this study are proposed as a simple screening tool to predict the risk of developing leg and foot ulcers in patients with DM.

## Introduction

Diabetes mellitus (DM), especially if uncontrolled, eventually leads to chronic complications with a significant proportion of patients having some complication even at the time of diagnosis [1]. Among the complications, diabetic ulcer disease has gained importance because of the socioeconomic burden it imposes on the patient, the patient’s family and on the health care system of the country. In UK and USA the prevalence of foot ulceration due to diabetes has found to be 5-7% [2]) and 6% [3] respectively, whereas in the developing countries it has shown a higher percentage [4,5]. In Sri Lanka, one third of type 2 diabetes patients have a risk of foot ulceration with some progressing to limb amputations [6].

Distal peripheral neuropathy (DPN) is reported to be an important cause of diabetic ulcer disease [7,8]. Although obesity, presence of hypertension, high serum cholesterol and alcohol consumption were identified as predictors of DPN in the developed region, in a national study including all provinces of Sri Lanka, these factor were not identified as predictors [9]. In this, which studied 528 subjects, rural residence and low household monthly income (<Sri Lankan Rupees (LKR) 12,000) revealed an association with DPN implying that the risk factors for foot ulcers in the developed world might be different to the Asian and South Asian region. In the South Asian region, poverty and poor educational level [8] contribute directly or indirectly to every health hazard. It may be that factors like inappropriate foot wear, duration of diabetes and poor glyceamic control might be more important in this region. 

The American Diabetes Association in its position statement - 2008 [10] recommends peripheral neuropathy screening at diagnosis of diabetes and at least annually thereafter, using simple clinical tests. Although DPN screening is a widely researched area, the findings show significant variation. Some authors have shown the effectiveness of single tests - assessment of the vibration sense [11], vibration perception threshold [12] and the pressure sensation using the 5.7/10g Semmes-Weinstein (SW) monofilament test [13], while others have shown the effectiveness of them when used in combination [14,15]. SW monofilament test is shown to be a simple, valid clinical tool for detecting neuropathy but requires a consensus on the protocol to be used [16] as the number of sites tested by monofilament vary widely between one site [15] up to ten [17,18]. However, none of these studies have shown these as predictors of foot ulcers, as diabetic ulcer disease seems to have a complicated aetiology, to which a number of structural, pathophysiological, behavioral and environmental factors contribute [19]. Longer duration of diabetes and poor level of education [8], prolonged hyperglycemia [20], inappropriate footwear [21], walking barefoot [22], weak pedal pulses [23], skin changes of the foot [21], presence of callus [24], Charcot deformity [25] and reduced ankle mobility [26] are some of the other common factors documented.

Due to devastating impact diabetic ulcers pose on the quality of life of the patients and the health care costs of a country, it is crucial to identify the at risk group for occurrence of ulcers, give them appropriate health education and monitor them closely with a view to prevent ulcer formation. Therefore, the aim of this study is to identify the socio demographic, life style and foot examination related predictors with a view to develop a simple and cost effective screening tool which is appropriate to be practiced in the outpatient settings. Urgency of this task becomes imperative with the increasing prevalence of DM globally. 

## Materials and Methods

### Ethics Statement

This study was approved by the Ethics review committees of University of Sri Jayewardenepura and Colombo South Teaching Hospital (CSTH).

Data were collected from type 2 DM subjects attending the Out Patient Department of the CSTH, Sri Lanka. Eighty eight subjects with leg and foot ulcers and 80 non ulcer controls were studied. Previously diagnosed DM subjects were identified and the diagnosis was confirmed by medical records. Subjects consenting to the study were recruited on a consecutive basis. The nature of the study was explained to the subjects prior to obtaining written informed consent. Subjects with leg and foot ulcers with an ulcer size not less than 2.5 cm^2^, Wagner scale two (scale 2 = ulcers extending into tendon, bone, or capsule) or three (scale 3 =deep ulcer with osteomyelitis, or abscess leg or foot ulcers ) and ulcer duration more than one week and less than six months were included in the study group. Subjects with cognitive impairment were excluded from both the study and control groups. Data on socio demographic factors (sex, age, education, household income) and diabetic and life style factors (duration of DM, mode of diagnosis, type of treatment, family history of DM, smoking, alcohol and type of footwear) were collected by an interviewer administered questionnaire. Physical examination of the lower limb was performed. Foot was examined for skin changes (dry/ cracked skin, skin discoloration and callus), and structural abnormalities (hammer toes, bunions and flat foot).

### Neuropathy assessment

Presence of DPN was assessed by testing the pressure sense, vibration sense and joint position sense. All tests were demonstrated to the subjects prior to the assessment of sensory modalities and they were performed while the subjects closed their eyes. 

Vibration sense was assessed by applying a 128-Hz vibrated tuning fork over the halluces. Subjects were asked to say ‘yes’ every time they perceived vibrations. If the subject was unable to perceive vibration sense while the examiner still perceives it, the test was recorded as abnormal. Test was repeated three times. If the subject did not indicate the result correctly for 2 or more times the test was reported as impaired.

Position sense was assessed by moving the great toe for 10° at inter phalangeal joint. Test was performed by flexing the great toe dorsally and ventrally while lightly grasping either sides with the thumb and the index finger of the investigator. Subjects were asked to indicate the position of the great toe as ‘up’ or ‘down’. If the subject was unable to indicate the direction of the great toe correctly, the test was recorded as abnormal. Test was repeated three times. If the test was abnormal for 2 or more times, it was reported as impaired.

Pressure sensation was assessed by applying the SW monofilament at ten sites (9 sites on the plantar surface of the foot; first toe, third toe, fifth toe, first metatarsal head, third metatarsal head, fifth metatarsal head, medial mid foot, lateral mid foot and heel, and 1 site on the dorsal surface between the base of the first and the second toe). Monofilament was pressed perpendicularly to the test site till it buckled and the pressure was exerted for two seconds. Subjects were asked to say ‘yes’ every time they perceive the pressure sensation at each site and test was repeated three times. At a given site, if the subject did not feel the sensation for 2 or more times the test was reported as abnormal. The pressure sensation was considered impaired, if the test was abnormal at any one or more sites.

### Statistical analysis

We compared differences of cases and controls using univariate analysis and calculated the odds ratios (ORs) and 95% CIs for socio demographic factors, diabetes and life style factors and findings of foot examination. Then multivariate analysis by logistic regression was performed to find significant variables for the final model for screening for foot ulcers. Data analysis was performed using statistical package for social sciences (SPSS) software (Version 19). 95% confidence was used for the determination of significance of probabilities.

## Results

Socio demographic, life style and foot examination related factors were compared in subjects with leg and foot ulcers and non-ulcer controls. In the univariate analysis, male sex, an education level of grade 6 and below, a monthly household income of LKR 50,000 and less were risk factors for foot ulcers in patients with DM (Table 1). A statistically significant increased risk was also observed for family history of DM and wearing slippers as opposed to sandals and covered shoes, whereas a decreased risk for foot ulcers was observed for diabetes diagnosed incidentally (OR - 0.25, 95% CI; 0.11 - 0.59) (Table 2). No consistent significant trend was observed with either smoking or alcohol. Table 3 shows the outcome of the examination of the foot. Intact vibration sense, position sense and a normal pressure sensation indicated by overall monofilament test results were statistically significant protective factors for foot ulceration. The presence of pressure sensation at first, third and fifth toes, first, third and fifth metatarsal heads, medial and lateral mid foot and dorsum was a protective factor for foot ulceration. However, presence of normal monofilament test at heel was not noteworthy.

**Table 1:** Socio demographic factors.

Category	Subcategory	Cases (%)	Controls (%)	OR	95% CI
Sex	Male	43 (48.9)	25 (31.2)	2.1	1.11 - 3.91
	Female	45 (51.1)	55 (68.8)		
Age (years)	≤ 50	28 (31.8)	18 (22.5)	1.6	0.81 - 3.2
	> 50	60 (68.2)	62 (77.5)		
Education	Grade 6 and below	22 (25.0)	6 (7.5)	4.1	1.57 - 10.76
	Grade 7 and above	66 (75.0)	74 (92.5)		
Monthly household income (LKR)	< 15,000	43 (48.9)	20 (25.0)	19.4	4.09 - 91.55
	15,000 - 50,000	43 (48.9)	42 (52.5)	9.2	2.06 - 42.20
	> 50,000	2 (2.3)	18 (22.5)	-	-

OR: odds ratio CI: confidence interval LKR: Sri Lankan rupees

**Table 2:** Diabetic and life style factors.

Category	Subcategory	Cases (%)	Controls (%)	OR	95% CI
Duration (years)	≤ 20	73 (83.0)	70 (87.5)	0.7	0.29 - 1.65
	> 20	15 (17.0)	10 (12.5)		
Mode of diagnosis	Incidental	35 (39.8)	10 (12.5)	0.25	**0.11 - 0.59**
	Screening	20 (22.7)	33 (41.2)	1.47	0.71 - 3.04
	Symptomatic	33 (37.5)	37 (46.2)	-	-
Type of treatment	Diet	7 (8.0)	1 (1.2)	0.24	0.02 - 3.01
	Diet and OHA	52 (59.1)	61 (76.2)	1.96	0.45 - 8.58
	Diet and OHA and Insulin	24 (27.3)	15 (18.8)	1.04	0.22 - 5.00
	Diet and Insulin	5 (5.7)	3 (3.8)	-	-
Family history of DM	Yes	51 (58.0)	26 (32.9)	2.8	**1.49 - 5.28**
	No	37 (42.0)	53 (67.1)		
Smoking	Current smoker	6 (14.3)	2 (7.4)	3.66	0.59 - 22.7
	Ex-smoker	27 (64.3)	14 (51.9)	2.36	0.79 - 7.02
	Never smoked	9 (21.4)	11 (40.7)	-	-
Alcohol	Current drinker	6 (14.3)	4 (15.4)	3.66	0.59 - 22.7
	Ex- drinker	23 (54.8)	14 (53.8)	2.36	0.79 - 7.02
	Never drank	9 (21.4)	5 (19.2)	-	-
	Social drinker	4 (9.5)	3 (11.5)	-	-
Type of footwear	Slippers	65 (74.7)	42 (55.3)	3.48	**1.38 - 8.72**
	Sandals	14 (16.1)	16 (21.1)	1.96	0.65 - 5.91
	Covered shoes	8 (9.2)	18 (23.7)	-	-

OHA: Oral hypoglycemic agents OR: odds ratio CI: confidence interval

**Table 3:** Examination of the foot.

Category	Sub category	Cases (%)	Controls (%)	OR (95% CI)
Healthy skin	Yes	35 (39.8)	43 (53.8)	0.59 (0.31 - 1.05)
	No	53 (60.2)	37 (46.2)	
Skin dry / cracked	Yes	33 (37.5)	35 (43.8)	0.77 (0.42 - 1.43)
	No	55 (62.5)	45 (56.2)	
Skin discoloration	Yes	26 (29.5)	3 (3.8)	10.7 **(3.11 - 7.23)**
	No	62 (70.5)	77 (96.2)	
Callus	Yes	23 (26.1)	15 (18.8)	1.5 (0.74 - 3.20)
	No	65 (73.9)	65 (81.2)	
Hammer toes	Yes	14 (15.9)	16 (20.0)	0.76 (0.34 - 1.67)
	No	74 (84.1)	64 (80.0)	
Bunions	Yes	14 (15.9)	11 (13.8)	1.19 (0.51 - 2.79)
	No	74 (84.1)	69 (86.2)	
Flat foot	Yes	8 (9.1)	1 (1.2)	7.9 (0.97 - 64.64)
	No	80 (90.9)	79 (98.8)	
Vibration sense	Intact	56 (63.6)	71 (88.8)	0.22 **(0.10 - 0.50)**
	Impaired	32 (36.4)	9 (11.2)	
Position sense	Intact	73 (84.9)	76 (95.0)	0.30 **(0.09 - 0.94)**
	Impaired	13 (15.1)	4 (5.0)	
Pressure sense (overall monofilament test)				
	Normal	27 (31.0)	51 (63.8)	0.26 **(0.13 - 0.49)**
	Abnormal	60 (69.0)	29 (36.2)	
Monofilament test of ten sites*				
No. 1	Normal	30 (35.7)	61 (76.2)	0.17 **(0.08 - 0.34)**
	Abnormal	54 (64.3)	19 (23.8)	
No. 2	Normal	40 (46.5)	68 (86.1)	0.14 **(0.06 - 0.32)**
	Abnormal	46 (53.5)	11 (13.9)	
No. 3	Normal	55 (64.0)	67 (83.8)	0.34 **(0.16 - 0.72)**
	Abnormal	31 (36.0)	13 (16.2)	
No. 4	Normal	47 (55.3)	61 (76.2)	0.38 **(0.19 - 0.75)**
	Abnormal	38 (44.7)	19 (23.8)	
No. 5	Normal	55 (65.5)	66 (82.5)	0.40 **(0.19 - 0.84)**
	Abnormal	29 (34.5)	14 (17.5)	
No. 6	Normal	59 (71.1)	72 (90.0)	0.27 **(0.11 - 0.65)**
	Abnormal	24 (28.9)	8 (10.0)	
No.7	Normal	69 (79.3)	74 (92.5)	0.31 **(0.12 - 0.83)**
	Abnormal	18 (20.7)	6 (7.5)	
No. 8	Normal	68 (79.1)	75 (93.8)	0.25 **(0.09 - 0.72)**
	Abnormal	18 (20.9)	5 (6.2)	
No. 9	Normal	68 (78.2)	62 (77.5)	1.03 (0.50 - 2.16)
	Abnormal	19 (21.8)	18 (22.5)	
No.10	Normal	60 (69.0)	75 (93.8)	0.15 **(0.05 - 0.41)**
	Abnormal	27 (31.0)	5 (6.2)	

OR: odds ratio CI: confidence interval

* Sites: 1. First toe; 2. Second toe; 3.Third toe; 4. First metatarsal head; 5. Third metatarsal head; 6. Fifth metatarsal head; 7. Medial mid foot; 8. Lateral mid foot; 9.Heel; 10. Dorsum

In the multivariate analysis, significant risk factors identified were education of grade 6 and below, a monthly household income of less than LKR 15,000 (US$ 140), impaired vibration sense, abnormal monofilament test on first toe, third toe, and fifth toe. The significant protective factors were incidental diagnosis of diabetes, wearing covered shoes and a normal monofilament test on first metatarsal head (Table 4). 

**Table 4:** Adjusted OR with significant variables for the final model for screening for foot ulcers.

Variables for screening	Adjusted OR	95% CI
Education of grade 6 and below	1.41	1.03 - 4.68
Income less than LKR 15,000 (US$ 140)	23.3	1.5 - 34.0
Incidental diagnosis of DM	0.03	0.003 - 0.28
Wearing covered shoes	0.003	0.00 - 0.28
Normal skin color	0.01	0.001 - 0.14
Impaired vibration sense	24.798	9.3 - 66.2
Abnormal monofilament test on 1^st^ toe	1.69	1.36 - 16.6
Abnormal monofilament test on 3^rd^ toe	3.4	1.1 - 10.6
Abnormal monofilament test on 5^th^ toe	1.8	1.61 - 12.6
Normal monofilament test on 1^st^ metatarsal head	0.10	0.00 - 0.67

OR: odds ratio CI: confidence interval LKR: Sri Lankan rupees

## Discussion and Conclusions

This study is the only reported study in Sri Lanka investigating the risk factors for diabetic foot ulcers. The objective of the heath care team should extend beyond treating the ulcers, and attempts should be taken to prevent ulcers in diabetic patients. Screening to identify factors which can accurately predict those who are at risk for foot ulceration is practical in an outpatient setting in a low resource country. Since most of these factors are modifiable, identification of these will allow prevention of ulcers in patients with DM. Due to the rapid increase in prevalence of DM in Sri Lanka, the patient turnover at diabetic clinics has substantially increased over the past decade, and thus the detection of these factors should be by a simple, less time consuming tool which is preferebly suitable to be administered even by a paramedical personnel. 

Male sex was one potential risk factor identified in the univariate analysis and this finding is consistent with studies elsewhere [8]. High prevalence of DM in men compared to women [27] and trauma prone occupations in males especially in low income groups may be contributing to this difference. Although the presence of a family history is a known etiological factor for DM, we did not find it as an independent risk factor for the development of diabetic ulcers. 

Ten factors were found to be significantly associated with the occurrence of ulcers in patients with diabetes in the multivariate analysis. Subjects with low level of education were found to have a higher risk of developing ulcers possibly due to the less likelihood of seeking treatment and the interest in life style adjustments. In fact low level of education has been suggested for increasing prevalence of DM in the rural sector of Sri Lanka [28], and DPN in UAE [8] both contributing to foot ulceration. A family monthly household income of less than LKR 15000 was another factor identified in this study which increases the risk of developing an ulcer (OR: 23.3; 95% CI 1.5-34.0). This finding is supported by a national study showing that DPN is associated with residence in rural areas and with a low household income [9] suggesting that the extreme poor may be having less opportunity for health services. Although the association of the durations of DM and DPN were reported previously [8,29-32], our findings are not in agreement probably because the true duration might be much longer than the duration calculated from the time of diagnosis. However, we found that the diagnosis of DM being incidental as a protective factor against ulcer formation. We also found that wearing slippers as opposed to sandals and covered shoes increases the risk of foot ulceration about 3-4 times. This is supported by a previous finding suggesting the use of appropriate footwear and wearing them indoors as well as outdoors to prevent foot ulcers [22].

In general examination of the foot, skin discoloration was found to be the only significant predictor of foot ulceration in our study population. Plantar callus [24] and hammer/claw toe deformity [25] were reported to be strong predictors of foot ulceration in previous studies investigating 63 and 749 DM patients respectively. However, we did not find any association with these probably due to differences in walking habits of study populations. 

Although DPN is reported to be the predominant root cause of diabetic foot ulceration, the diagnosis is complicated and requires the assessment of multiple features [21]. Different symptom scores have being used extensively in previous studies [31-33]. We did not advocate them because neuropathic sensory symptoms do not always accurately detect DPN in patients with type 2 DM [34] and sometimes they are subjective and correlate poorly with other tests [35]. Instead, our aim was to test for three sensory modalities using basic clinical skills and simple equipment with a view to find their suitability in identifying DM patients at risk for foot ulceration. Although impairment of all three sensations appeared to be significant risk factors for ulceration in the univariate analysis, only the impairment of vibration and pressure sensations were significant in the final model. Several previous reports have shown the value of using 128-Hz tuning fork alone [11] or in combination with 10g SW monofilament test [36]. In relation to our findings, impaired vibration sense is a very strong predictor of diabetic ulceration (OR: 24.7; 95% CI 9.3-66.2). The use of SW monofilament test is found to be an ideal, sensitive, easily learned, simple, inexpensive and less time consuming screening tool to assess DPN [17,36,37]. Although various combination of sites are suggested [17,18,37,38], we found that the presence of an abnormal monofilament test on 1^st^ ,3^rd^,and 5^th^ toes significantly increases the ulcer risk by 1.69,3.4 and 1.8 fold respectively recommending these 3 out of 10 sites to be used in screening. The finding of a normal monofilament test on the first metatarsal head strengthens the predictive value of the screening tool as it is found be an independent protective factor. This is in agreement with the suggestion of including 1^st^ metatarsal head as a common site of screening in previous studies [18,36]. Although nerve conduction studies are the gold standard to detect DPN, they are not cost effective to be used in a low resource country as ours and further, they are impractical in a primary care setting. 

In summary, we have identified 10 independent risk and protective factors which can be used in a simple screening tool suitable for screening all DM patients to predict the risk of developing leg and foot ulcers. However, the possibility that ulceration may have influenced the occurrence of some of the factors identified in the tool is a limitation which has to be resolved by a prospective study. Training the staff to administer the tool is suggested to minimize the inter-observer variability. Although glycaemic control is known to reduce the incidence of DPN, it was not considered due to practical limitations in outpatient settings. Future study is planned to validate this tool for our population.
